# Achievements in Applications of Antioxidants and Bioactive Compounds in Food: From Agriculture to Health Benefits

**DOI:** 10.3390/antiox13101247

**Published:** 2024-10-16

**Authors:** Mia Kurek, Frédéric Debeaufort, Andrée Voilley

**Affiliations:** 1 Laboratory for Food Packaging, Faculty of Food Technology and Biotechnology, University of Zagreb, 10000 Zagreb, Croatia; 2 Department BioEngineering, Institut Universitaire de Technologie IUT-Dijon, University of Burgundy, Blvd Dr. Petitjean, CEDEX, 21078 Dijon, France; frederic.debeaufort@u-bourgogne.fr; 3 UMR PAM 1517-PCAV (Physical-Chemistry of Food and Wine Laboratory), Université Bourgogne Franche-Comté, L’InstitutAGro, Université de Bourgogne, INRAE, Esplanade Erasme, 21000 Dijon, France; andree.voilley@u-bourgogne.fr

Natural foods and food components are becoming increasingly popular worldwide because people are concerned about eating healthy diets. Natural additives outperform their artificial counterparts, which are limited due to their association with many chronic diseases. The bioactive compounds found in a variety of plants, spices, superfoods, and other similar items are frequently linked to the health-promoting qualities of natural ingredients. Waste from the agrifood sector has become an important focus in scientific circles, with the goal of following circular economy principles to reduce waste and conserve resources by closing resource, material, and energy loops. Modern agriculture produces significant amounts of waste that end up in landfills, resulting in controversial consequences instead of being reintroduced into the production chain for new purposes. It is often emphasized that a circular bioeconomic approach is relevant for the agrifood sector because this sector significantly contributes to greenhouse gas emissions, water and energy use, and the degradation of natural ecosystems. The reutilization of these compounds has numerous potential uses, including in nutraceuticals, cosmetics, functional foods, food and feed additives, and more. In addition to being environmentally friendly, this leads to the creation of products with added value. Bioactive phytochemicals with antioxidant, anti-inflammatory, hypolipidemic, and antibacterial properties can be found in waste materials, such as residues and byproducts. As people become more concerned about healthy diets and natural additives, this trend is driven by the availability of numerous nutrients. However, the stability, delivery, and bioavailability of these compounds in the human body after digestion are limited by a few obstacles. Therefore, in this Special Issue, we explore the antioxidant properties and processing potential of various medicinal herbs and underutilized bioresources for developing value-added products or products related to cosmetic, pharmaceutical, or other food and agricultural applications, such as packaging. These products could be used in other systems that are able to enhance their bioavailability.

Even though agriculture is very important in Mediterranean countries, the olive industry is a good example of a target sector that can contribute. Indeed, the olive industry is especially important from a socioeconomic, cultural, and dietary standpoint. According to [1], implementing waste and by-product valorization strategies for olive by-products can encourage the development of novel methods within the most influential socioeconomic conditions pertaining to the olive sector. In this context, Contribution 1 focused on using two Tunisian local olive cultivars to obtain olive leaf extracts rich in bioactive compounds. The extracts were tested to reduce the acrylamide concentration in Californian-style black olives. Enhancements were seen in terms of phenol content and antioxidant characteristics, without negatively affecting the sensory characteristics of treated olives. Additionally, research has shown that extracts can partially break down acrylamide during digestion, thereby lessening its impact on the digestive system. However, it appears that the addition of the extract had little effect on its digestion in the gastrointestinal system. Studies have emphasized the significance of using by-products to produce high-quality goods with added value in the transition to a more circular and sustainable economic model.

Given that various plant sources, such as roots, flowers, leaves, and so on, have a significant impact on the composition of plant extracts, the specific extraction conditions and methods are crucial for their further use. Contribution 2 developed an enzymatic extraction method using an experimental design that combined a surface-response Box–Behnken design for optimization with a screening Plackett–Burman design for determining factors with higher significance. Their study demonstrated that enzymatic extraction was suitable for producing antioxidant-rich extracts, with high levels of repeatability and intermediate precision. Considering the temperatures, amount of organic solvents, and processing time, this method can be considered even greener than conventional extraction methods, such as ultrasound- or microwave-assisted extractions.

A wide variety of phytochemicals, polyphenols, alkaloids, terpenes, and saponins are included in the category of bioactive compounds. Antioxidant, anticarcinogenic, antiallergenic, anti-inflammatory, antimutagenic, and antimicrobial properties are among their remarkable biological effects. The study of phenolic compounds is essential for their characterizing and maximizing the potential of this valuable plant resource. Studies published in this Special Issue highlight research on moringa (*Moringa oleifera* Lam) (Contribution 2), Dunal (Ashwagandha) (*Withania somnifera* L.) (Contribution 3), pepper (*Capsicum annuum* L.) (Contribution 4), *Brassica rapa* (Contribution 5), *Raphanus species* (Contribution 6), and chokeberry (*Aronia melanocarpa*) (Contribution 7). Apart from providing antioxidant activity data, each study focuses on specific targets.

Contribution 2 showed that using a mix of pectinases from *Aspergillus niger* was efficient for extracting quercetin 3-*O*-glucoside, quercetin malonyl glucoside, quercetin hydroxy methyl glutaroyl glucoside, quercetin acetyl glucoside, kaempferol-3-glucoside, and isorhamnetin-3-glucoside from moringa. The produced extracts exhibited great antioxidant properties. In another study, aqueous Ashwagandha extracts presented higher antioxidant activity than hydromethanolic ones, with higher polyphenol and ascorbic acid contents (Contribution 3). The potential anti-proliferative activity of pepper fruits from diverse varieties with different capsaicin contents (California < Piquillo < Padrón < Alegría riojana) against several tumor cell lines (lung, melanoma, hepatoma, colon, breast, pancreas, and prostate) was investigated in Contribution 4. Authors focused on the link between capsaicin content and anti-proliferative activity against tumor cell lines. The authors showed that bioactive compounds from capsaicin could serve to combat tumor cell lines from the liver (Hep-G2) and pancreas (MIA PaCa-2), thus addressing human health issues. In Contribution 5, authors showed that mustard plants rich in glucosinolates (subspecies of *Brassica rapa*: *B. rapa* subsp. trilocularis and *B. rapa* subsp. Chinensis) can help treat excessive oxidative stress and inflammatory responses associated with the development of various diseases, including cancer. Therefore, this plant, with enhanced antioxidant capacity, may be considered a beneficial daily vegetable that can reduce the risk of inflammation-associated diseases. In Contribution 7, the identification of compounds primarily responsible for the antioxidant properties of aronia berry extracts was done. Radical-scavenging properties and the content levels of chlorogenic acids was found to be strongly correlated to unripe green chokeberry fruits.

Due to their poor water solubility, susceptibility to chemical damage, and decreased effectiveness when taken orally, many bioactive functional ingredients have low bioavailability. Whey protein from goat milk was shown to be effective in the creation of nano-delivery systems (Contribution 8). The stability of developed nanoparticles against aggregation has garnered significant attention due to its potential applicability in acidic to neutral food products. The stability of these nanoparticles during digestion in the stomach makes it easier for them to pass into the small intestine, where they can be absorbed. This has the potential to boost intestinal absorption, bioavailability, and therapeutic efficacy.

In order to protect packaged food and bioactive ingredients, there is a trend toward developing new active antioxidant packaging systems that provide additional protection against oxidation. In another study (Contribution 9), new functionalized antioxidant coatings on polylactic acid films were applied and tested as potential materials for use with food sensitive to oxidation.

Finally, in this Special Issue, one review article summarized the properties and potential uses of *Cytisus* plants as an underexploited bioresource of bioactive compounds for developing new food ingredients (antioxidants, preservatives, additives, etc.), nutraceuticals, and even direct therapeutic agents (anticancer, antibacterial, etc.) (Contribution 10).

In summary, this Special Issue focused on scientific papers addressing various complementary studies aimed at enhancing the potential functionalities of bioactive compounds found in unexploited agrifood sources and their by-products that may benefit human health. This Special Issue also featured topics related to global waste reduction, considering food and health-related issues and packaging.
